# Editorial: Advanced green polymers for medical purpose – trends and challenges in the circular economy

**DOI:** 10.3389/fbioe.2024.1511632

**Published:** 2024-11-13

**Authors:** Joanna Rydz, Marta Musioł, Barbara Zawidlak-Węgrzyńska, Kristof Molnar

**Affiliations:** ^1^ Centre of Polymer and Carbon Materials Polish Academy of Sciences, Zabrze, Poland; ^2^ Department of Chemistry, Faculty of Medicine in Zabrze, Academy of Silesia, Katowice, Poland; ^3^ Department of Biophysics and Radiation Biology, Faculty of Medicine, Semmelweis University, Budapest, Hungary; ^4^ Department of Food, Agricultural and Biological Engineering, College of Food, Agricultural, and Environmental Sciences, The Ohio State University, Wooster, OH, United States

**Keywords:** bio-based polymer, (bio)degradable polymer, biocomposite, biomaterial, biopolymer, material structure and property, circularity

Nowadays, a very important impetus for the development of new functional materials is not only their performance but also whether they are environmentally friendly. It caused the development of bioplastics – bio-based and/or (bio)degradable alternatives to traditional plastics – crucial for sustainable development and environmental protection. Their application in medical devices and their packaging, presents new challenges regarding biodegradability and bioconversion, pushing the medical sector towards a more sustainable, circular economy. Green polymers, derived from renewable resources and possessing biodegradable properties, offer an economically viable solution, aiding in waste reduction and promoting sustainable life cycles (Islam et al., 2024; Sikorska et al., 2024).

Research into sustainable bioplastics is crucial for advancing materials science and fostering technological innovations, as it drives the development of polymers that are safe for people and the environment. Given the low rates of reuse and recycling of polymer materials and the limited demand for recycled plastics, the European strategy on plastics aims to align EU legislation with circular economy principles, emphasizing the necessary actions for national, regional authorities, and industries to promote sustainability and environmental responsibility in material development (Moshood et al., 2022; Musioł et al., 2024).

The purpose of the Research Topic was to provide a contemporary overview of the latest developments in the field of advanced, resource-efficient, eco-friendly, and sustainable next-generation bioplastics for the closed-loop economy. Papers accepted under this Research Topic addressed interdisciplinary approaches aimed at the development of (bio)degradable and/or renewable polymer materials for sustainable medical industry needs.

To enhance the biocompatibility of brain implants and mitigate the damage they cause to surrounding tissue, researchers are exploring flexible materials that better match the mechanical properties of brain tissue, thus reducing friction and inflammation. Innovations in soft robotics, (bio)degradable materials, and advanced coatings are being developed to create implants that not only adhere more effectively to brain tissue but also integrate seamlessly with the biological environment (Qi et al., 2023). Darlot et al. conducted a brief biocompatibility assessment of the NeuroSnooper intra-cortical implants, which feature a microelectrode array constructed from a flexible polymer-metal-polymer stack with microwires designed to resemble axons. For implantation, they were integrated into (bio)degradable needles made of poly(lactic-*co*-glycolic acid) (PLGA), highlighting their potential integration into neural tissue.

While bone grafting procedures are pivotal in enhancing dental implant success and addressing craniofacial defects, challenges such as limited availability of donor tissue, potential for infection, immune responses, and variability in bone integration can complicate outcomes. Additionally, the risk of complications, underscores the importance of selecting appropriate grafting materials and methods tailored to individual patient needs. As advancements continue, ongoing research aims to mitigate these drawbacks while improving the efficiency and effectiveness of bone grafting in dental procedures (Zhao et al., 2021). The development by Feroz et al. of a novel biomimetic dual-layered keratin/hydroxyapatite scaffold using an iterative freeze-drying technique, coupled with an ionic liquid-based green method for keratin extraction, showcases an innovative approach in bone tissue engineering. *In vitro* studies indicate that these scaffolds possess significant potential, in bone regeneration and repair. This Research Topic has been covered extensively by Xing et al. in Mini Review.

The increasing use of human body implants, presents challenges such as infection risk due to their foreign nature. Research is actively focused on creating antibacterial materials that can be integrated into these, ultimately reducing the incidence of infections associated with long-term implant use (Haq and Krukiewicz, 2023). The study of Meng et al. highlights that 10 nm nanosilver particles demonstrate significant antibacterial activity through multiple mechanisms, including the disruption of bacterial cell membranes and walls, which ultimately leads to bacteria DNA damage. This approach underlines the potential of nanosilver particles as effective agents against bacterial infections.

The rising prevalence of infertility Research Topic has led to a significant increase in the demand for assisted reproductive technologies (Lazzari et al., 2023). Belda Perez et al. propose that utilizing extrusion-based 3D printing of polycaprolactone (PCL) offers a promising approach for creating innovative *in vitro* fertilization devices, which may support oviductal epithelial cells and thereby improve the development of bovine embryos in reproductive technologies.

The proposed scope concerned research on the development and manufacturing of innovative, technologically advanced materials that have general applicability and that form the basis for evolving knowledge on this Research Topic. Particular emphasis was placed on environmental-friendly materials, with a short global carbon life cycle, and/or suitable for recycling. Combining these with physicochemical studies was to fill a gap in existing knowledge and allow for the design and identification of new resource-efficient, environmentally safe polymer materials for the circular economy. Original Research articles and Mini Review are an attempt to cover aspects of the current trends and help the expansion of such materials.
